# Cone beam computed tomography vs. Periapical Radiograph: Diagnostic accuracy in endo and periodontal lesions

**DOI:** 10.1590/0103-644020256704

**Published:** 2026-02-02

**Authors:** Maria Caroline Rios Piecha, Fernanda Geraldo Pappen, Fábio de Almeida Gomes, Camila Silveira Sfreddo, Natália Marcumini Pola

**Affiliations:** 1 Graduate Program in Dentistry, Universidade Federal de Pelotas, Pelotas, Brazil; 2 Semiology and Clinics Department, Faculty of Dentistry, Universidade Federal de Pelotas, Pelotas, Rio Grande do Sul, Brazil; 3 Health Science Center, Universidade de Fortaleza, Fortaleza, Brazil

**Keywords:** diagnostic imaging, endodontics, endo-periodontal lesions, cone-beam computed tomography

## Abstract

Objectives: This study aimed to compare the diagnostic accuracy of dental specialists when assessing endodontic and periodontal lesions using periapical radiography (PR) alone and PR combined with cone-beam computed tomography (PR+CBCT). Methods: In this cross-sectional study, 30 endodontists and 30 periodontists evaluated ten clinical cases involving endodontic and periodontal tissues at two time points. The cases included vertical root fractures/fissures, endodontic perforation, persistent apical periodontitis, concomitant endo-periodontal injury, and localized periodontitis. Initially, participants reviewed clinical data and periapical radiographs for each case, formulating a diagnostic hypothesis via a structured questionnaire. Subsequently, CBCT images were introduced, and participants reassessed the images and reached a diagnosis again. The correct diagnosis, as established by the dentist responsible for each case, was included as one of the answer choices. Diagnostic accuracy for overall and specific lesion types after incorporating CBCT was analyzed using the McNemar test, with statistical significance set at 5%. Results: The overall percentage of correct diagnoses was 67.2% for PR+CBCT and 42.2% for PR. PR+CBCT provided accurate diagnoses in 58.8% of cases for both specialties. Regarding lesion type, PR+CBCT correctly identified a greater number of endo-periodontal lesions with and without root damage compared to PR alone. Conclusions: PR+CBCT significantly improves diagnostic accuracy for both endodontists and periodontists in cases involving both pulp and periodontal tissues.

## Resumo

Objetivos: Este estudo teve como objetivo comparar a acurácia diagnóstica de especialistas em Odontologia na avaliação de lesões endodônticas e periodontais utilizando exclusivamente radiografia periapical (RP) e a combinação de radiografia periapical com tomografia computadorizada de feixe cônico (RP+TCFC). Métodos: Em um estudo transversal, 30 endodontistas e 30 periodontistas avaliaram dez casos clínicos envolvendo tecidos endodônticos e periodontais em dois momentos distintos. Os casos incluíram fraturas/fissuras radiculares verticais, perfuração endodôntica, periodontite apical persistente, lesão endo-periodontal concomitante e periodontite localizada. Inicialmente, os participantes analisaram os dados clínicos e as radiografias periapicais de cada caso, formulando uma hipótese diagnóstica por meio de um questionário estruturado. Posteriormente, as imagens de TCFC foram disponibilizadas, e os participantes reavaliaram os casos, emitindo novamente um diagnóstico. O diagnóstico considerado correto, estabelecido pelo cirurgião-dentista responsável por cada caso, foi incluído entre as alternativas de resposta. A acurácia diagnóstica global e por tipo de lesão após a introdução da TCFC foi analisada pelo teste de McNemar, adotando-se nível de significância de 5%. Resultados: A porcentagem global de diagnósticos corretos foi de 67,2% para RP+TCFC e 42,2% para RP isolada. A combinação RP+TCFC resultou em acurácia de 58,8% em ambos os grupos de especialistas. Em relação ao tipo de lesão, RP+TCFC possibilitou a identificação correta de um número maior de lesões endo-periodontais com e sem comprometimento radicular quando comparada à RP isolada. Conclusões: O uso combinado de RP+TCFC aumenta significativamente a precisão diagnóstica tanto de endodontistas quanto de periodontistas em casos que envolvem simultaneamente os tecidos pulpar e periodontal.

**Figure:**
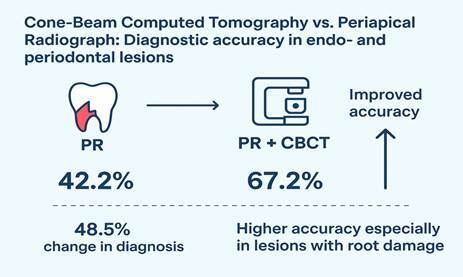

## Introduction

Accurate diagnosis is fundamental for developing an appropriate treatment plan and ensuring predictable outcomes in complex dental conditions. Inaccurate diagnostic assessment may lead to inappropriate treatment, unnecessary costs for the patient, uncertain prognoses, and even tooth loss ^1^ ^,^ ^2^. The initial diagnostic process can be particularly challenging because it requires integration of clinical findings with radiographic interpretation and depends significantly on the clinician's experience ^3^.

A significant diagnostic difficulty lies in distinguishing between endodontic and periodontal lesions ^4^. Anatomically, these tissues are interconnected through three main pathways: dentinal tubules, lateral or accessory canals, and the apical foramen-the main bidirectional route for the spread of inflammatory agents and microorganisms ^5^. External factors such as carious lesions, fractures, resorptions, and perforations may further increase communication between the pulp and periodontium ^6^. When periapical and periodontal alterations coexist, evaluating their potential interrelationship becomes essential for determining an accurate diagnosis ^7^.

According to the 2018 World Workshop on the Classification of Periodontal and Peri-Implant Diseases and Conditions, endo-periodontal lesions (EPLs) are chronic conditions classified according to the presence or absence of root damage ^8^ ^,^ ^9^. This approach reflects clinically relevant differences in pathogenesis, prognosis, and therapeutic management. Accurate identification of EPLs, therefore, depends on comprehensive clinical and radiographic assessment and, in many cases, a collaborative approach between endodontists and periodontists ^10^.

Periapical radiographs (PR) remain the most common imaging tool in endodontic and periodontal diagnosis; however, their two-dimensional nature limits the ability to visualize complex structures and overlapping anatomical details ^11^. Cone-beam computed tomography (CBCT) offers three-dimensional imaging and multiplanar reconstruction, improving visualization of root morphology, bone defects, and lesion extent ^2^ ^,^ ^12^ ^,^ ^13^ ^,^ ^14^ ^,^ ^15^ ^,^ ^16^. Nonetheless, its use should be limited to cases of higher diagnostic complexity, as recommended by professional associations such as the AAE and AAOMR, due to additional ionizing radiation exposure ^17^.

Several studies have confirmed the superior diagnostic performance of CBCT compared with PR in detecting endodontic and endo-periodontal lesions involving root damage ^1^ ^,^ ^3^ ^,^ ^14^. However, the diagnostic contribution of CBCT in non-root-damage endo-periodontal lesions, and its comparative usefulness for endodontists and periodontists, remains underexplored. The use of CBCT in complex cases has been shown to significantly influence treatment planning among endodontists, with reported decision changes ranging from 52.9% to 63.3% ^11^ ^,^ ^12^ ^,^ ^14^. Given the prevalence of complex cases in clinical practice, understanding how CBCT affects diagnostic accuracy for both specialties is clinically relevant ^10^ ^,^ ^18^ ^,^ ^19^ ^,^ ^20^.

Therefore, the aim of this study was to compare the diagnostic accuracy of dental specialists when assessing endodontic and periodontal lesions using periapical radiography (PR) alone and PR combined with cone-beam computed tomography (PR+CBCT). The null hypothesis was that the addition of CBCT imaging would not significantly alter diagnostic accuracy in cases involving endodontic and periodontal lesions.

## Materials and methods

### Study design and Participants' Informed Consent

This cross-sectional study adheres to the guidelines in the Strengthening the Reporting of Observational Studies in Epidemiology (STROBE) checklist ^21^. Approval was obtained from the Institutional Research Ethics Committee (protocol #54290221.8.0000.5318), and all participants provided written consent.

### Sample selection

The sample included dentists with postgraduate degrees or actively registered specialists in Endodontics or Periodontics, working in private practices, public health facilities, or educational institutions across Rio Grande do Sul, Brazil. Recruitment was conducted via email or telephone. General practitioners, practitioners holding certifications in both endodontics and periodontics, and participants who did not complete both phases of evaluation (PR and PR+CBCT) were not eligible for inclusion.

The sample size was estimated based on a prior study that reported the average difference observed in clinical decision-making by endodontists, with or without the use of CBCT ^(^ ^19^. The following parameters were considered: a 5% standard error, 80% power, 95% confidence level, a difference of 31.3% reported in the follow-up of tooth extraction cases in teeth with moderate endodontic impairment, and 34.1% for cases with complex endodontic impairment. This yielded a minimum sample size of 60 participants, divided into two groups: Endodontists (n=30) and Periodontists (n=30).

### Case Selection

Ten clinical cases were selected, including instances of vertical root fractures/fissures ^4^, endodontic perforation ^1^, persistent apical periodontitis ^2^, concomitant endo-periodontal injury ^2^, and localized periodontitis ^1^. Patient confidentiality was maintained.

For each case, clinical data such as primary complaint, symptoms, cervical or periapical fistula, pulp sensitivity, apical palpation, and percussion test responses were provided. Additional details on previous endodontic treatment, presence of intraradicular posts, crown restorations, and clinical periodontal parameters were included. Finally, periapical radiographs and tomographic sections (axial, sagittal, and/or coronal) of the respective tooth were included to complete the case presentation. All radiographic and tomographic images were obtained from real patient cases previously treated at the dental clinics of the authors’ affiliated institutions. PRs were captured using digital sensors under standardized exposure conditions (RX Digital Sensor Fit HD 2.0 Acteon, Micro Imagem, Indaiatuba, SP-Brazil; and RX device DABI ATLANTE Spectro 70x RX, Ribeirão Preto, SP-Brazil).

CBCT images were acquired using a Morita Veraview X800 (J. Morita Tokyo MFG. Corp., Tokyo, Japan) unit, a small-volume CBCT scanner, with voxel sizes ranging from 0.08 mm to 0.125 mm and fields of view between 4×4 cm and 5×5 cm. These settings enabled high-resolution imaging of localized regions, particularly relevant for detecting root fractures and periapical or periodontal lesions. To ensure consistency and optimal visualization, images were edited using Adobe Photoshop (Adobe, San Jose, CA, USA) to unify brightness, contrast, and orientation. PRs were adjusted to fixed grayscale ranges (Input Levels: 20/1.00/235) and exported as TIFFs. Slice selection aimed to ensure standardized and transparent visualization of the region of interest for all participants, avoiding ambiguity in lesion depiction. All slices were extracted from real patient cases without altering diagnostic content, ensuring consistency across evaluations.

The "correct diagnosis" used in the data analysis corresponded to the reference standard, which was defined according to the lesion type and established through clinical, surgical, or histopathological confirmation, rather than relying solely on the treating dentist's judgment. Specifically: (a) for root fractures or perforations, the reference standard was surgical visualization; (b) for apical periodontitis, it was histopathological examination in surgically treated cases or clinical and radiographic follow-up of 12 months confirming post-treatment healing; and (c) for periodontitis, the reference standard consisted of clinical periodontal parameters such as probing depth, clinical attachment level, and radiographic bone loss.

### Study Protocol

Each participant attended an individual, in-person session with a trained researcher. Sociodemographic and professional information was collected, including gender, skin color, area of specialization, time of professional experience, public or private university graduation, and professional practice location. All case evaluations were conducted on a tablet (Galaxy Tab A 2020, Samsung Brazil) and performed directly in Google Workspace, where periapical and tomographic images were embedded in a structured online questionnaire. Participants viewed each case in sequence and recorded their diagnostic hypothesis through the same digital form. No external image-viewing software or calibration parameters were required, and standardized written instructions were provided to ensure uniformity across all evaluations.

**Figure 1:**
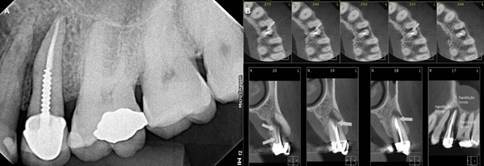
Example of one of the clinical cases. (A) Periapical radiograph of tooth 25. (B) CT scans provided for this case.

In the initial questionnaire, participants were presented with clinical data and periapical radiographs sequentially. They recorded their diagnostic hypotheses by selecting from four predefined options. In the second questionnaire, participants reevaluated the same cases, this time with the addition of tomographic images alongside the periapical radiographs and provided updated diagnoses (Figure 1). All radiographic and tomographic images used in this study were original files provided by the imaging clinic, without any editing, annotation, or arrows. This ensured that the evaluations reflected real clinical conditions, as routinely performed by professionals in practice. The correct diagnosis, as established by the dentist responsible for each case, was included as one of the answer choices. The diagnosis was recorded as correct when the specialist selected the option that corresponded to the diagnosis provided by the responsible dentist. All clinical case evaluations were conducted on the same day.

### Data Analysis

Data analysis was conducted using Stata software (StataCorp, 2014. Stata Statistical Software: Release 14.1. College Station, TX: StataCorp LP). The independent variable was the imaging modality (periapical radiography alone [PR] versus periapical radiography combined with cone-beam computed tomography [PR+CBCT]). The dependent variable was the diagnostic accuracy (correct or incorrect diagnosis). Categorical variables were summarized by frequency distributions. The McNemar test was used to compare diagnostic accuracy between PR and PR+CBCT, with a 5% significance level. In addition, subgroup analyses were performed according to: (a) the type of endo-periodontal lesion, classified as with or without root damage; and (b) the specialty of the examiner (endodontist or periodontist).

For analytical purposes, the cases were stratified into two groups: Group 1 - endo-periodontal lesions with root damage (including fractures, cracks, and perforations) and Group 2 - endo-periodontal lesions without root damage (including concomitant endo-periodontal lesions, solely endodontic, and solely periodontal conditions). This stratification was based on the current classification proposed by the 2018 World Workshop on the Classification of Periodontal and Peri-Implant Diseases and Conditions ^9^, which emphasizes the clinical relevance of distinguishing lesions by the presence or absence of root structural involvement due to its impact on prognosis and treatment planning.

## Results

### Sociodemographic and Professional Data

Sociodemographic and academic characteristics are presented in Table 1, with most participants being female, of white ethnicity, educated at public institutions, and working in private practice. The sample consisted of 60 specialists: 30 periodontists and 30 endodontists. Each of the 60 participants evaluated all 10 cases twice: first with PR alone, then with PR+CBCT. This yielded 1,200 total assessments (600 per modality).

**Table 1:** Distribution of the sociodemographic and professional data of the sample (n=60).

		N	%
Gender	*Female*	41	68.3
	*Male*	19	31.6
Skin color	*White*	55	91.6
	*Non-white*	5	8.3
Specialization	*Endodontics*	30	50.0
	*Periodontics*	30	50.0
Time of experience	*<5 years*	23	38.3
	*5-10 years*	19	31.6
	*>10 years*	18	30.0
Graduate	*Public*	47	78.3
	*Private*	13	21.6
Professional practice location	*Educational Institution*	26	43.3
	*Private Service*	46	76.6
	*Public Service*	12	20.0

### Analysis of Diagnostic Accuracy and Imaging Modalities

In 48.5% of cases (n=291), the diagnosis changed after using the combined PR+CBCT approach. This combination resulted in a significantly higher rate of correct diagnoses compared to PR alone (67.2% vs. 42.2%, p<0.01). Among cases correctly diagnosed with PR, 78.7% remained accurate with PR+CBCT, while 58.8% of all PR+CBCT diagnoses were accurate (Table 2). In endo-periodontal lesions with root damage, PR+CBCT corrected 64.4% of the initial PR misdiagnoses and achieved consistent accuracy in 66.3% of cases. In endo-periodontal lesions without root damage, accuracy remained the same in 85.7% of cases, and PR+CBCT corrected 50.4% of the errors from PR alone (Table 3).

When analyzed separately for each lesion type, diagnostic performance varied between conditions. Sensitivity ranged from 55.0% (endodontic perforation) to 86.7% (localized periodontitis), whereas specificity remained consistently high (74.2-89.6%).

The highest diagnostic accuracy was observed for localized periodontitis (89.3%), followed by vertical root fractures/fissures (83.0%). These findings indicate that the combined use of periapical radiography and CBCT enhances clinicians’ ability to correctly identify complex endo-periodontal lesions, particularly those involving root structural damage (Table S1).

**Table 2:** Comparison of diagnostic success and error rates between PR and PR+CBCT (n=1200).

Periapical Radiography	Periapical Radiography + CBCT*		Total
	Success n (%)	Error sn (%)	
Success n (%)	199 (78.7)	54 (21.3)	253
Errors n (%)	204 (58.8)	143 (41.2)	347
Total	403	197	600

*Statistically significant differences with CBCT (*p*< 0.01; McNemar's test).

**Table 3:** Percentage of correct and incorrect diagnoses across different lesion groups (n=1200).

Type of lesion*	Periapical Radiography n (%)	Periapical Radiography + CBCT**		Total
		Success n (%)	Errors n (%)	
Group 1 (root damage)	Success	61 (66.3)	31 (33.7)	92
	Errors	134 (64.4)	74 (35.6)	208
	Total	195	105	300
Group 2 (no root damage)	Success	138 (85.7)	23 (14.3)	161
	Errors	70 (50.4)	69 (49.6)	139
	Total	208	92	300

* Group 1= Endo-periodontal lesions with root damage (fractures/cracks and perforations); Group 2 = Endo-periodontal lesions without root damage (concomitant endoperiodontal lesions, and independent endodontic/periodontal lesions).

**Statistically significant differences with CBCT (*p*< 0.01; McNemar's test).

### Comparison of Diagnostic Accuracy Between Endodontists and Periodontists

**Figure 2:**
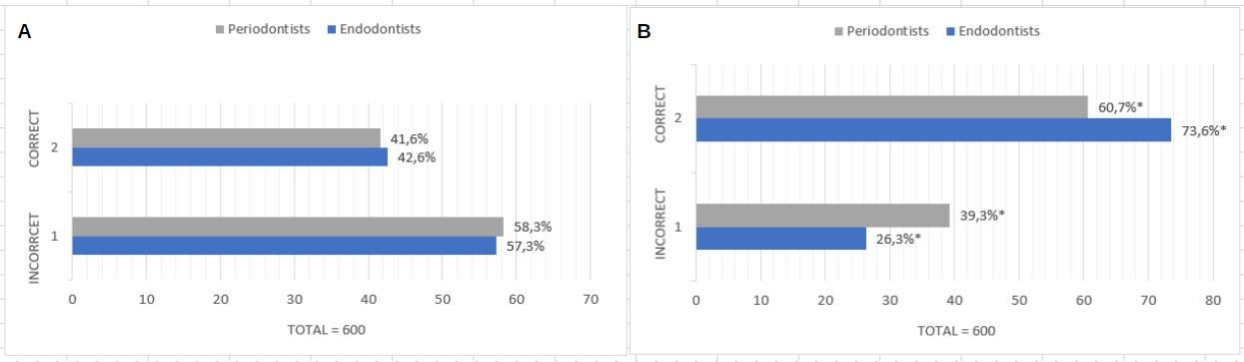
Correct and incorrect diagnosis rates between endodontists and periodontists. (A) Correct and incorrect rates between endodontists and periodontists using periapical radiography (n=600). (B) Correct and incorrect rates between endodontists and periodontists using PR+CBCT (n=600). Statistically significant differences in the percentage of correct and incorrect diagnoses after using the tomographic method (*P< 0.01; McNemar's test).

No significant difference was observed between specialists’ diagnoses using PR (p=0.804). However, the association of CBCT increased diagnostic accuracy for both groups, with endodontists achieving higher accuracy (73.6%) than periodontists (60.7%, p<0.01) (Figure 2).

## Discussion

Endo-periodontal disorders present significant clinical challenges and demand a multidisciplinary approach for proper management ^(^ ^9^. In this context, CBCT is widely recognized as an important tool for case planning, though limited research has explored its specific impact on diagnostic decision-making ^(^ ^11^ ^,^ ^14^. This study investigated how the PR+CBCT influences the diagnosis of lesions affecting the pulp and periodontium among endodontists and periodontists, showing that this association significantly improved diagnostic confidence across specialties.

The current literature extensively documents the substantial utility of CBCT as a diagnostic and planning tool in teeth with endodontic involvement ^11^ ^,^ ^12^ ^,^ ^14^ ^,^ ^15^ ^,^ ^16^ ^,^ ^19^ ^,^ ^20^. This is attributable to the capability of computed tomography to manipulate images, exporting them in Digital Imaging and Communications in Medicine (DICOM) format for analysis in various software platforms, facilitating detailed three-dimensional assessment of the affected tooth. A cross-sectional study examined patients with endodontically compromised teeth and determined the diagnosis of lesions with or without the inclusion of tomographic examination. Following tomographic assessment, diagnoses were altered in 35% of the evaluated sample ^(^ ^16^. These findings were subsequently supported by another study that used CBCT in the preoperative evaluation of endodontically compromised teeth, revealing modifications in 19% of pulp diagnosis and 30% of responses concerning apical changes compared to the initial clinical assessment. This underscores CBCT's significant role in elucidating the etiology of endodontic pathologies and guiding treatment decisions ^(^ ^1^. The present study supports these findings, revealing that CBCT contributed to both diagnosis changes and correct diagnoses when assessing pulp and periodontal lesions.

The highest percentage of correct diagnoses occurred when radiographic and tomographic data were available, suggesting that tomographic imaging enhances diagnostic accuracy in endo-periodontal lesions. Additionally, clinical data, especially periodontal parameters, helped guide professionals more effectively than radiographic images alone. Similar studies have focused primarily on imaging alone ^12^ ^,^ ^15^ ^,^ ^16^. Dias et al. ^20^ conducted a study involving 85 teeth suspected of root fractures by two radiologists, incorporating clinical data followed by periapical radiographs and CBCT. They found that CBCT exhibited higher diagnostic accuracy (65.6%) compared to PR (40.5%), underscoring the importance of integrating periodontal assessment and bone level information with imaging exams for enhanced diagnostic success.

Regarding lesion types, professionals achieved over twice the correct diagnoses for endo-periodontal lesions with root damage (Group 1) when using PR+CBCT (p < 0.01). Recent studies emphasize the difficulty in diagnosing and treating vertical root fractures and fissures, lesions that are increasingly prevalent ^22^ ^,^ ^23^ ^,^ ^24^. Radiographs may inadequately capture these situations, depending on the location and extent of the fissure or fracture and the limited detail radiographs provide ^24^ ^,^ ^25^. CBCT has been reported to be 4.4 times more effective than periapical radiographs for detecting bone defects associated with longitudinal fractures. Using CBCT, it is possible to differentiate these injuries: cracked teeth typically show intact cortical bone and angular defects near fissures, whereas vertical root fractures present J-shaped defects, probing depths greater than 6 mm, and cortical bone loss ^(^ ^24^. Although no significant differences emerged between specialists’ interpretations, both groups performed better using PR+CBCT.

The diagnostic metrics confirm that PR + CBCT significantly improves clinicians’ ability to identify root fractures and complex endo-periodontal conditions.

While specificity remained stable across lesion types, sensitivity markedly increased for root fractures and concomitant endo-periodontal lesions. The moderate sensitivity for endodontic perforations likely reflects the low number and subtle presentation of such cases, which inherently limit detection accuracy. Overall, these findings align with prior reports highlighting CBCT's superior diagnostic utility for endodontically involved teeth ^11^ ^,^ ^20^.

It is noteworthy that there was a considerable percentage of errors across both imaging modalities. This outcome may be due to the challenge participants faced in diagnosing cases without direct patient evaluation. Despite detailed clinical descriptions, individual approaches to case analysis may have varied, and some participants might have encountered case-specific questions that the provided data did not fully address. Discrepancies were also observed between the two imaging methods in endo-periodontal lesions without root damage, with a significant percentage of incorrect diagnoses for both PR and PR+CBCT (49.6%). It is important to consider that localized periodontitis and combined endo-periodontal lesions are typically considered moderate to low complexity, where CBCT is not routinely used. Consequently, the additional data from CBCT may have led to some confusion among participants.

This study has some limitations. First, the selection of CT scans lacked standardization and was based solely on the region of interest. The timing of additional exams also varied between cases. Despite this, all radiographic and CT scans were digital and of high quality. The cases were chosen to reflect common endodontic lesions; however, their uneven distribution reflects real-world clinical prevalence and the challenges inherent in diagnostic confirmation. While this variability may limit generalizability, subgroup analyses highlight the importance of CBCT in complex lesions, such as root fractures. The use of ten cases limits the breadth of clinical scenarios represented and may affect external generalizability. This number was selected to provide a representative case mix while maintaining feasible session length and participant engagement. Furthermore, some clinical details, such as prior history of periodontitis, time since initial endodontic treatment, information on adjacent teeth, occlusal data, and clinical photographs, were not available, which may have influenced the accuracy of participants' diagnostic decisions. Although diagnostic accuracy was assessed by the percentage of correct responses, a method commonly used in similar studies ^1^ ^,^ ^20^, it was not possible to calculate traditional diagnostic metrics such as sensitivity, specificity, or overall accuracy. This limitation stems from the study design, which presented only one correct diagnostic alternative for each case, making it impossible to construct the 2x2 contingency tables required for these analyses. We recognize that such metrics can provide valuable insights into false-positive and false-negative rates. Therefore, this methodological limitation will be addressed in future studies by adopting a design that allows for the calculation of these diagnostic indicators.

To the best of our knowledge, this is the first study to evaluate the impact of PR combined with CBCT on diagnosing lesions involving both pulp and periodontal tissues by specialists from both fields. Previous studies have primarily focused on endodontists ^1^ ^,^ ^16^ ^,^ ^17^ ^,^ ^19^ ^,^ ^23^ ^),^ underscoring the importance of collaboration between these two specialties for managing complex cases, leading to more accurate diagnoses and, subsequently, clearer prognoses and treatment plans. Additionally, face-to-face sessions in a single session ensured sample consistency, with no dropouts, and the same imaging tool for all participants minimized discrepancies in image quality and visibility.

Within the limits of this study, the conclusion is that PR combined with CBCT improves diagnostic accuracy for both endodontists and periodontists in cases involving both pulp and periodontal tissues.

**Table S1:** Diagnostic performance (sensitivity, specificity, and accuracy) by lesion type. PR + CBCT

Lesion type	Sensitivity(%) [95% CI]	Specificity (%)	Accuracy (%)
Vertical root fracture/fissure	79.2 [±5.1]	85.6	83.0
Endodontic perforation	55.0 [±12.6]	83.3	80.5
Persistent apical periodontitis	71.7 [±8.1]	77.1	76.0
Concomitant endo-periodontal lesion	75.0 [±7.7]	74.2	74.3
Localized periodontitis	86.7 [±8.6]	89.6	89.3

## Data Availability

The research data are available within the article
